# Establishment of clinical predictive model based on the study of influence factors in patients with colorectal polyps

**DOI:** 10.3389/fsurg.2023.1077175

**Published:** 2023-02-23

**Authors:** Yu Huang, Yating Liu, Xu Yin, Tianpeng Zhang, Yaoguang Hao, Pengfei Zhang, Yang Yang, Zhihan Gao, Siyu Liu, Suyang Yu, Hongyan Li, Guiying Wang

**Affiliations:** ^1^ Department of Gastrointestinal Surgery, The Third Hospital of Hebei Medical University, Shijiazhuang, China; ^2^ Department of Second Anorectal, Shijiazhuang Hospital of Traditional Chinese Medicine, Shijiazhuang, China

**Keywords:** intestinal polyps, influencing factors, intestinal tumor, nomogram, prevention

## Abstract

**Background:**

Colorectal cancer (CRC) is the most common gastrointestinal malignancy and is generally thought to be caused by the transformation of colorectal polyps. It has been shown that early detection and removal of colorectal polyps may reduce the mortality and morbidity of colorectal cancer.

**Objective:**

Based on the risk factors associated with colorectal polyps, an individualized clinical prediction model was built to predict and evaluate the possibility of developing colorectal polyp.

**Methods:**

A case-control study was conducted. Clinical data were collected from 475 patients who underwent colonoscopy at the Third Hospital of Hebei Medical University from 2020 to 2021. All clinical data were then divided into training sets and validation sets by using R software (7:3). A multivariate logistic analysis was performed to identify the factors associated with colorectal polyps according to the training set, and a predictive nomogram was created by R software based on the multivariate analysis. The results were internally validated by receiver operating characteristic (ROC) curves, calibration curves, and externally validated by validation sets.

**Results:**

Multivariate logistic regression analysis showed that age (OR = 1.047, 95% CI = 1.029–1.065), history of cystic polyp (OR = 7.596, 95% CI = 0.976–59.129), and history of colorectal diverticulums (OR = 2.548, 95% CI = 1.209–5.366) were independent risk factors for colorectal polyps. History of constipation (OR = 0.457, 95% CI = 0.268–0.799) and fruit consumption (OR = 0.613, 95% CI 0.350–1.037) were protective factors for colorectal polyps. The nomogram demonstrated good accuracy for predicting colorectal polyps, with both C index and AUC being 0.747 (95% CI = 0.692–0.801). The calibration curves showed good agreement between the predicted risk by the nomogram and real outcomes. Both internal and external validation of the model showed good results.

**Conclusion:**

In our study, the nomogram prediction model is reliable and accurate, which can help early clinical screening of patients with high-risk colorectal polyps, improve polyp detection rate, and reduce the incidence of colorectal cancer (CRC).

## Introduction

Colorectal polyps are a general term for all neoplasm that protrudes from the intestine. The CP has a high incidence worldwide, with a detection rate of 10%–20% through colonoscopy (1, 2). According to the well-known adenoma-to-carcinoma sequence; colorectal adenomatous polyps may develope into colorectal cancer owing to a series of genetic and epigenetic abnormalities (3).The majority of CRCs originate from adenomatous polyps (adenomas), which usually take more than a decade to become malignant(4, 5). Colorectal cancer (CRC) is the third most common cancer in the world, but it is also the second most common cause of death among the diseases (6). This poses a serious threat to human health. For this reason, the early identification and treatment of colorectal polyps have become increasingly important.

In most cases, colorectal polyps are asymptomatic Most of the patients seek medical attention due to the corresponding symptoms or abnormal physical examination results (such as fecal occult blood), and polyps are later revealed by colonoscopy. Colonoscopy is the most effective method for detecting colorectal cancers and polyps (7). In addition to detecting colorectal polyps, colonoscopy can also perform therapeutic polypectomy, making it the preferred method to identify and treat polyps. It would be very helpful if we encourage high-risk patients to have colonoscopy screenings in order to detect and treat colorectal polyps early. Several studies have shown that colonoscopy can significantly reduce colorectal cancer incidence and mortality in a variety of countries (8, 9). By intervening in the early stages of colorectal disease, adenomatous polyps can be prevented from transforming into colorectal cancer. Colonoscopy screening for CRC and adenoma, on the other hand, is rather expensive and unavailable in rural areas with limited resources in China. What's more, the rate of utilization and compliance of colonoscopies have remained relatively low in China due to their invasive nature and cumbersome preparation for insertion (10, 11). Moreover, the incidence of colorectal polyps and CRC has been on the rise in the past few years, with a trend of younger patients (12). However, colonoscopy is not a screening procedure, and young people are less inclined to undergo it.

Thus, it is necessary to correctly identify the high-risk population of colorectal polyps and promote colonoscopy in a wider range of potential patients, which can improve screening efficiency, reduce medical costs and save medical resources. In our study, risk factors for colorectal polyp incidence would be analyzed in order to establish a nomogram that can evaluate the risk of colorectal polyp incidence. With this model, we manage to assess the patients' risk of colon polyps objectively, screen for colorectal polyps in high-risk populations, with an individual and intuitive solution, as well as educate the public about colorectal polyps.

## Materials and methods

### Study population

We conducted a case-control study in this research. Clinical data of patients who underwent colonoscopy at the Gastrointestinal Surgery Department of the Third Hospital of Hebei Medical University from January 2020 to September 2021 were collected as subjects. The data collected from clinical studies were retrospectively analyzed. The following criteria were used to collect clinical case information: inclusion criteria: (1) Patient undergoing colorectal polypectomy and electronic colonoscopy at the Third Hospital of Hebei Medical University. (2) Colonoscopy can access to the ileocaecal region. (3)Intestinal preparation was perfect, and mucosal observation was unaffected after endoscopy. (4) Patients who had an indication for a colonoscopy. (5) Patients who were able to receive telephone follow-up were included. On the other hand, the exclusion criteria were as follows: (1) with symptoms of mental illness. (2) Patients who were not compliant or refused to participate in the study were excluded. (3) Patients with pathologically confirmed or previous colorectal cancer. (4) Patients who had previously undergone colorectal resection. This study adhered to the principles and ethical requirements of the Helsinki Declaration. Due to the retrospective nature of the study, the requirement for informed patient consent was waived.

### Data collection

Based on the criteria above, data were collected from 475 patients, in which including 298 patients with colorectal polyps (CP group) and 177 patients with normal endoscopic and inflammatory lesions (control group). Polyploidy lesions were found during endoscopic surgery, and the pathology was non-neoplastic lesions, while in the control group, there were normal or inflammatory lesions. Data included family history, relative diseases, personal diet habits, and pathological results were collected.

### Results of evaluation

Colonoscopy was performed by 2 experienced gastroenterologists who performed at least 1000 colonoscopies per year and were blinded to the bowel preparation regimens the patients received. Endoscopic diagnosis of colorectal polyps would be based on Classification and Digestive Neoplasm (2019 edition) (13). The pathological diagnosis of the colorectal polyp was made by two pathologists. If the diagnosis was disputed, further discussion would be held, or a third pathologist experienced in explaining the results will be invited. Still, if there was disagreement, the seriousness of the disease would prevail.

### Statistical analysis

The data was processed and statistical analyses were conducted using SPSS 25.0 (SPSS, Inc., Chicago, IL, United States), and R software (version 4.0.1) with the ‘rms’ package. The quantitative data that conform to the normal distribution were expressed as means ± standard deviation, and the difference between groups was checked by an independent sample t-test. Comparisons between groups that did not obey the normal distribution were done using the nonparametric test. Data from the enumeration process were expressed as percentages and cases, and the Chi-square test was used to determine whether groups differed.

Randomly, we divided the 475 patients into training sets and validation sets according to the proportions of 7:3. The training set included 332 people, while the verification set included 143 people. Based on a single factor logistic regression analysis of the training data, the potential risk factors for colorectal polyps were identified. In univariate analysis, exposure factors with *P* ≤ 0.2 were selected (14–16). In order to examine the independent risk factors for colorectal polyp development, these potential risk factors were included in a multivariate logistic regression analysis.

The independent risk factors were incorporated into R software and created the rosette map using the ‘rms’ program package to predict colorectal polyp risk. Bootstrapping was applied to repeat the sampling 1000 times to conduct internal verification of the nomogram, and validation set data was applied for external verification. The c-index and area under Receiver Operating Characteristic (AUC) curves were used to measure the discrimination of the nomogram, and the calibration curve between the predicted and observed probabilities was used to evaluate the calibration. Last but not least, the validation set data was applied to validate the model externally. A nomogram chart's clinical application value was assessed by evaluating its sensitivity, specificity, predictive value, and likelihood ratio of the optimal cut-off. The optimal cut-off value was determined by the Youden index. Statistically significant differences were considered to exist when the *p*-value is below 0.05 in all tests.

## Results

### Study baseline characteristics

In the training set and the validation set, there were no significant differences found in baseline characters (*P* > 0.05). The detailed results were shown in Table 1. According to colonoscopy, 204 (61.4%) and 94 (65.7%) patients were detected in the case and control groups, respectively.

**Table 1:** Comparison of baseline characteristics of subjects.

Variable		Training set (*n* = 332)	Validation set (*n* = 143)	t/*χ*2/Z	*P* value
Gender ([n (%)])				0.120	0.729
	Woman	138 (41.6)	57 (39.9)		
	Man	194 (58.4)	86 (60.1)		
Age (years,mean ± sd)		53.95 ± 14.92	53.43 ± 12.94	0.377	0.706
Height ([m,mean ± sd])		1.68 ± 0.07	1.67 ± 0.07	1.331	0.184
Weight ([kg,x ± s])		68.81 ± 12.45	68.49 ± 10.19	0.262	0.794
BMI ([kg/m2,x ± s])		24.24 ± 3.58	24.44 ± 3.00	−0.581	0.561

### Univariate logistic regression model analysis results of colorectal polyp occurrence

As part of the training set, 332 subjects were classified into the case (*n* = 204) and control (*n* = 128) groups Univariate logistic regression analysis showed that the occurrence of colorectal polyps may be correlated with gender, age, BMI, blood glucose, blood pressure, occupational habits (Brain/Physical), family genetic history, history of depression, history of cystic polyps, history of the colorectal diverticulum, history of diabetes, history of Non-Steroidal Anti-inflammatory Drugs (NSAIDs) drugs, and consumption of fruits and vegetables (*P* ≤ 0.2). The results were shown in Table 2.

**Table 2:** Single-factor logistic regression analysis based on training set.

Variable	Case (*n* = 128)	Control (*n* = 204)	OR (95% CI)	*P*
Gender(Man/woman)	67/61	127/77	1.502 (0.960–2.350)	0.075
Age(years,x ± s)	57.95 ± 13.78	47.57 ± 14.49	1.051 (1.034–1.069)	0.000
BMI ([kg/m2,x ± s])	24.57 ± 3.14	23.73 ± 4.15	1.072 (1.003–1.145)	0.039
Blood glucose(Yes/No)	41/163	13/115	2.225 (1.141–4.339)	0.019
Blood fat (Yes/No)	40/164	21/107	1.243 (0.695–2.223)	0.464
Blood pressure(Yes/No)	144/60	106/22	0.498 (0.288–0.863)	0.013
Degree of Education (above/below)	85/119	57/71	0.890 (0.570–1.390)	0.608
Career(Brain/Physical)	107/97	77/51	0.731 (0.467–1.144)	0.170
Family history (Yes/No) of Colorectal cancer	45/159	17/111	1.848 (1.006–3.395)	0.048
Depression(Yes/No)	13/191	13/115	0.602 (0.270–1.344)	0.215
Constipation (Yes/No)	45/159	48/80	0.472 (0.290–0.768)	0.003
Gastric polyps(Yes/No)	16/188	12/116	0.823 (0.376–1.801)	0.625
Gastroesophageal reflux (Yes/No)	47/157	36/92	0.765 (0.462–1.267)	0.298
Cystic polyps(Yes/No)	18/186	1/127	12.290 (1.620–93.231)	0.015
Cholecystolithiasis (Yes/No)	6/198	3/125	1.263 (0.310–5.140)	0.745
HP(Yes/No)	113/191	13/115	0.602 (0.270–1.344)	0.215
IBS (Yes/No)	13/191	7/121	1.177 (0.457–3.032)	0.736
Irritable bowel (Yes/No)	51/153	39/89	0.761 (0.465–1.244)	0.276
Proctiti (Yes/No)	28/176	18/110	0.972 (0.514–1.841)	0.931
Colorectal diverticulum(Yes/No)	44/160	11/117	2.925 (1.449–5.904)	0.003
Rheumatic immune disease (Yes/No)	10/194	9/119	1.467 (0.579–1.726)	0.419
Diabetes(Yes/No)	19/185	6/122	0.682 (0.269–5.377)	0.127
Statins (Yes/No)	28/176	15/113	0.834 (0.427–1.631)	0.596
NSAIDs(Yes/No)	28/176	10/118	0.533 (0.249–1.138)	0.104
Smoke (Yes/No)	63/141	33/95	0.777 (0.474–1.275)	0.319
Drink(Yes/No)	74/130	45/83	0.952 (0.600–1.511)	0.836
Breakfast (Occasionally/Often)	185/19	114/14	0.836 (0.404–1.733)	0.631
Greasy food(Occasionally/Often)	43/161	23/105	0.820 (0.567–1.440)	0.490
Double salt food (Occasionally/Often)	40/164	29/99	0.833 (0.486–1.428)	0.506
Meat(Occasionally/Often)	147/57	87/41	1.215 (0.751–1.996)	0.427
Fruits (Occasionally/Often)	136/68	101/27	0.535 (0.320–0.895)	0.017
Vegetables(Occasionally/Often)	134/70	94/34	0.692 (0.425–1.127)	0.139

### Colorectal polyps: multivariate logistic regression model analysis, independent risk factors

Due to the desire to analyze the factors related to the occurrence of colorectal polyps as much as possible, the index *P* ≤ 0.2 was analyzed by multiple factors. Multivariate logistic regression analysis showed that age (OR = 1.047, 95% CI = 1.029–1.065), history of cystic polyp (OR = 7.596, 95% CI = 0.976–59.129) and history of the colorectal diverticulum (OR = 2.548, 95% CI = 1.209–5.366, *P* < 0.05) were independent risk factors for colorectal polyps. History of constipation (OR = 0.457, 95% CI = 0.268–0.799) and fruit consumption (OR = 0.613, 95% CI = 0.350–1.037) were a protective factor for colorectal polyps. The detailed results were shown in Table 3. In addition, the Hosmer-Lemeshow goodness of fit test and the Omnibus test of model coefficients confirmed the reliability of the regression model.

**Table 3:** Multivariate analysis based on training set.

Variable	B	SE	Wald-χ	*P*	Adjusted OR	95% CI
Age	0.046	0.009	27.344	0.000	1.047	1.029–1.065
Constipation	−0.783	0.272	8.278	0.004	0.457	0.268–0.799
Cystic polyps	2.028	1.047	3.751	0.053	7.596	0.976–59.129
Colorectal diverticulum	0.935	0.38	6.053	0.014	2.548	1.209–5.366
Fruits	−0.489	0.285	2.937	0.087	0.613	0.350–1.037
Constant term	−1.588					

### Building a predictive nomogram model

**Figure 1:**
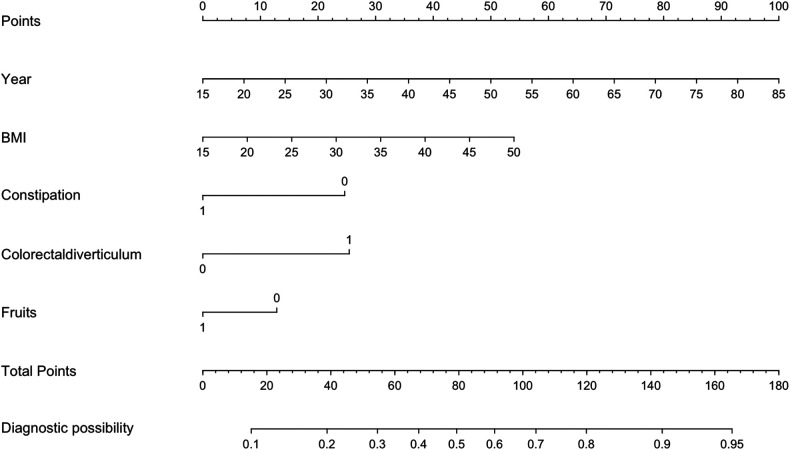
Nomogram predicting risk of colorectal polyps.

Based on the 5 independent predictors examined by multivariate logistic regression analysis, a risk nomogram for colorectal polyps was built (Figure 1): Points correspond to the upper rating scale for each independent predictor, and the total Points for each subject are the sum of the scores of each independent predictor. Colorectal polyp risk is determined by the value of the total score on the risk axis of colorectal polyps. The higher the total score, the higher the risk of colorectal polyps.

### The validation of a model includes both external and internal checks

**Figure 2:**
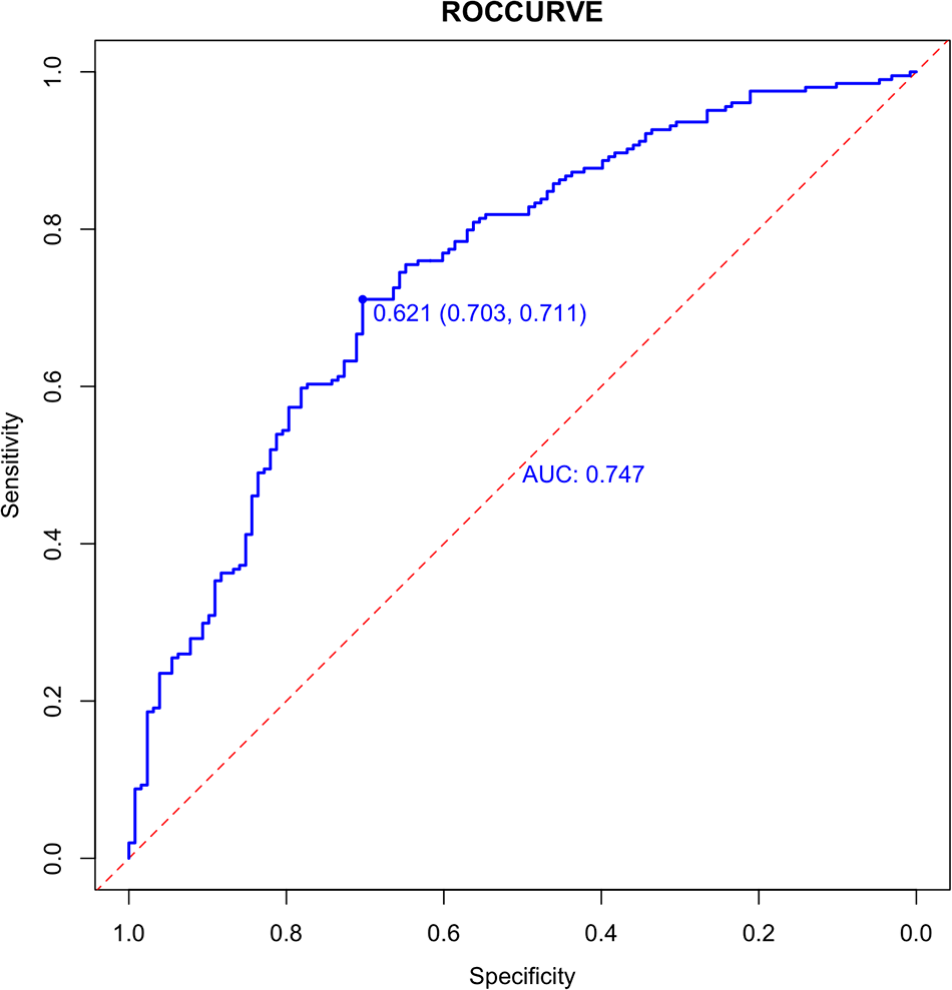
Internal validation of the column diagram: ROC. Of note: AUC: 0.747; 95% CI: 0.692-0.801; *p* < 0.001.

**Figure 3:**
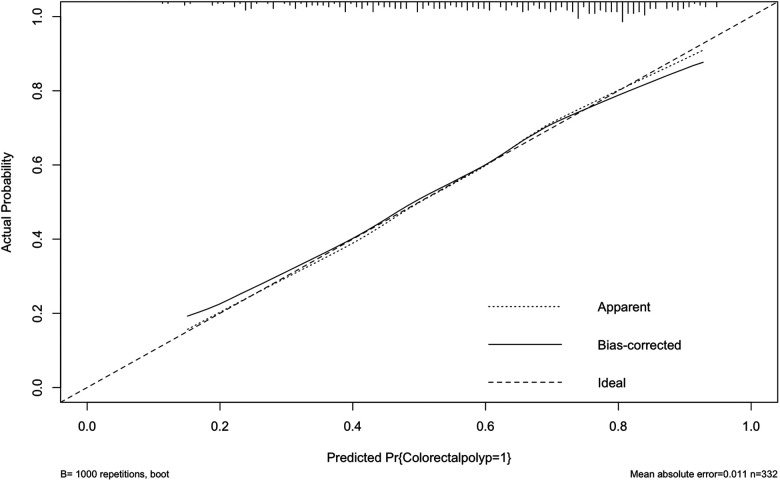
Internal validation of the column diagram: calibration curve of note: C index: 0.747.

Internally, the nomogram was verified by repeating sampling 1000 times using the Bootstrap method in R software, and verification sets from external sources served as the data for external verification. The nomogram had good segmentation and calibration in predicting colorectal polyps, with C index and AUC both of which was 0.747 (95% CI = 0.692 −0.801) (Figure 2). There was a good agreement between the nomogram prediction model and the colonoscopy detection of real wind risks in the calibration curve (Figure 3).

**Figure 4:**
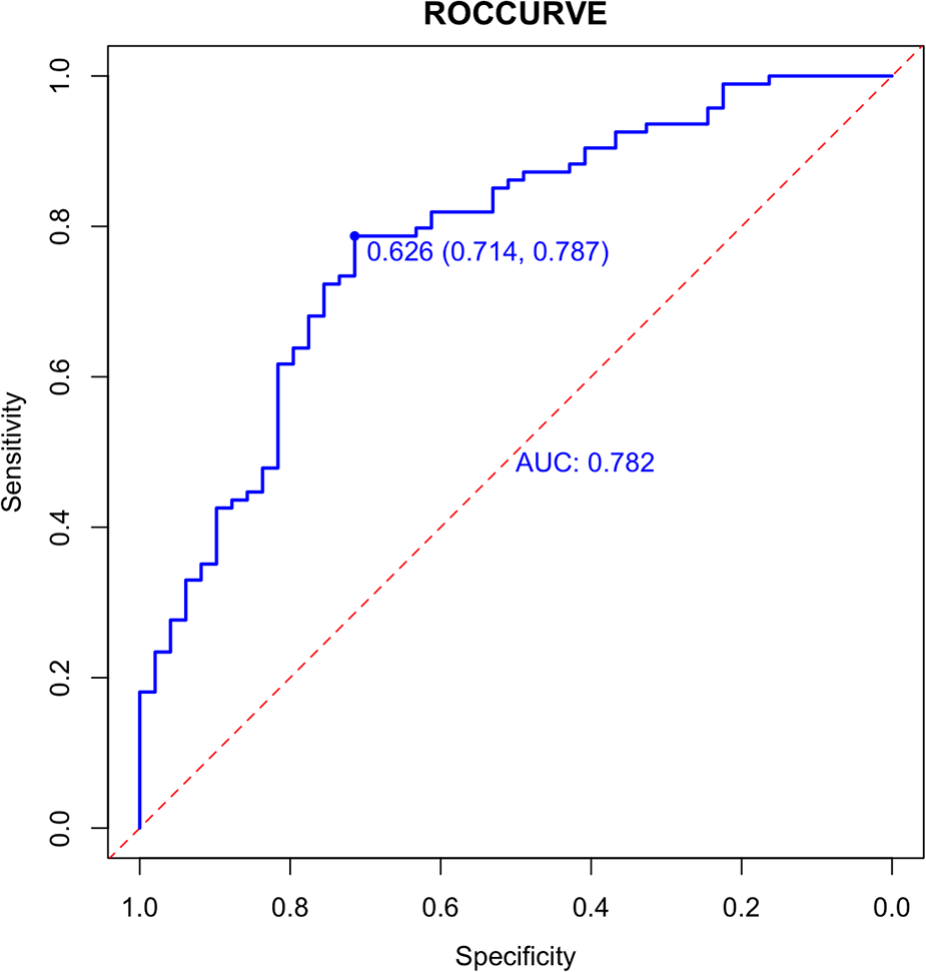
External validation of the column diagram: ROC. Of note: AUC: 0.782; 95% CI: 0.703-0.861; *p* < 0.001.

**Figure 5:**
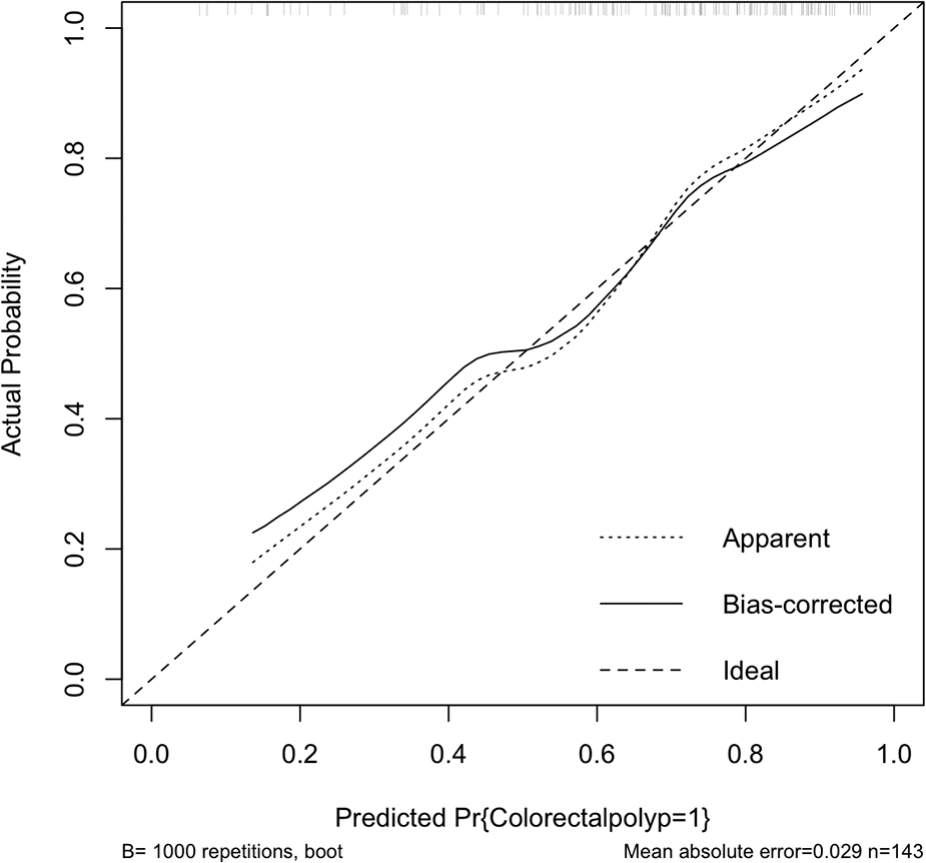
External validation of the column diagram: calibration curve. Of note: C index: 0.782.

In the validation set, external validation of the model was conducted. In the validation set, the graph also performed well in agreement and calibration, both C index and AUC were 0.782 (95% CI = 0.703–0.861) (Figure 4). A good calibration curve between the predicted and actual wind hazards can also be seen in (Figure 5).

### An analysis of the clinical effectiveness of the nomogram model

The optimal cut-off value of the training set's nomogram's total score was calculated based on the Youden index as about 93.8 points in order to determine the clinical significance of the model. Those whose overall score exceeded or equaled 93.8 were considered high-risk subjects, and those whose score was less than 93.8 were considered low-risk subjects. Under the cut-off value, sensitivity and specificity were 70.3% and 71.1% in the training set and 71.4% and 78.7% in the verification set.

## Disscussion

A colorectal polyp is an abnormal growth that sticks out from the colorectal surface. Generally, CRC develops from colorectal polyps (CP) when an adenoma-carcinoma sequence is initiated (3, 17). A colorectal polyp detected early and treated effectively can improve the outcome of colorectal cancer prevention and treatment. Colorectal cancer screening reports have shown that it reduces the risk of mortality; but patient adherence to screening recommendations remains low. The selection of CRC screening modality depends not only on the validity of the modality in the target population but also on the feasibility, affordability, compliance, and clinical capacity of screening, particularly in resource-limited settings (18). Furthermore, most participants with CRC do not have any clinical symptom, which complicates the process of finding intestinal polyps. In our study, we analyzed the risk factors for colorectal polyp and built an individualized clinical prediction model to help improve the efficiency of colorectal polyp screening and reduce the incidence of colorectal polyps.

Age and history of colorectal diverticulums were independent risk factors for colorectal cancer development in the study. Furthermore, constipation (Dehydrated stool and reduced frequency of stools) and vegetable consumption (eat vegetables regularly) were protective factors for colorectal polyps. In the Asia Pacific Colorectal Screening Scoring System (19) and its revisions (20), people at high risk for CRC and advanced adenomas are screened based on their age. At the same time, studies have found that colorectal adenoma incidence increases with age (21). In our study, this was corroborated.

In our study, a history of gallbladder polyps' disease was shown as an independent risk factor for developing CP. In a previous study performed in a Japanese case-control study, the prevalence of CP in patients with biliary tract disease was significantly higher than that of controls. The presence of biliary tract disease is an independent risk factor for colon polyps (OR = 1.57,95% CI = 1.14–2.18) (22). One possible explanation is that gallbladder disease produces more deoxygenated bile acids and this secondary bile acid can be formed by promoting the formation of intestinal adenoma (23).

Our data showed that the history of colorectal diverticulums was an independent risk factor for colorectal polyps, in addition to age and gallbladder polyps' disease, which is consistent with a previous study by Liu et.al (OR = 2.548, 95% CI = 1.209–5.366, *P* < 0.05) (24). Nevertheless, some researchers disagreed. Their argument was that there was controversy surrounding the link between colorectal diverticular disease and colorectal adenoma (25).

Further, our study found that the consumption of fruit and a history of constipation were protective factors for CPs. Previous studies have shown that smoking, alcohol consumption, high-fat diet, red meat, and low-fiber diets increase the risk of colorectal polyps (25–28). Smoking has also been shown to promote the development of cancer of the colon originating from colorectal polyps (29). The results of our study, however, suggested that fruit consumption alone was protective against colorectal polyps. In another aspect, constipation was associated with colorectal cancer (30, 31). There has not been a detailed analysis of the relationship between them and the mechanism of their mutual influence.

Many researches focuses on the development of clinical models of disease risk, and many related risk models are available, including those for coronary heart disease and colorectal cancer (32, 33). Currently, most colorectal disease predictive models are based on colorectal cancer (34, 35). Few colorectal polyp risk prediction models exist. A small number of studies developed predictive models was established based on postoperative pathological features combined with artificial intelligence (36, 37). While these studies differ from the research concept of this study, the findings of our study are more consistent with the concept of early diagnosis and early treatment in some way.

A nomogram was constructed based on the predictors screened by statistical analysis. In this study, a nomogram could predict risk with 0.703 accuracies, and 0.711 accuracies for the validation set. This nomogram can provide a certain reference value for medical workers to intuitively analyze individual risk, as well as for patients at high risk of colorectal polyps to be screened. Depending on the predicted risk, a reasonable evil screening procedure can be designed.

There are still some limitations in this study. The enrolled population selected in this study was a group of people who visited our hospital for colorectal examination at one time, and the bias of admission rate was inevitable in the selection of the population. Due to the different medical conditions of the population and the uneven understanding of colorectal polyps, the proportion of patients in the positive group may be too high. And the study sample was relatively small; it was only conducted retrospectively over one year at one hospital. In addition, the established model had a weaker generalization ability and transferability than the space test. It is still necessary to carry out large-scale research with multiple centers in order to verify this. Also screen the high-risk population for colorectal polyps, so as to achieve the goal of early detection, prevention, and treatment of colorectal polyps, as well as to serve as a reference for further prevention and treatment.

## Conclusions

In our study, the nomogram predictive model is reliable and accurate, which helps early clinical screening of patients with high-risk colorectal polyps, improves polyp detection rate, and reduces the incidence of colorectal cancer (CRC).

## Data Availability

The raw data supporting the conclusions of this article will be made available by the authors, without undue reservation.
